# Human cultured epidermis accelerates wound healing regardless of its viability in a diabetic mouse model

**DOI:** 10.1371/journal.pone.0237985

**Published:** 2020-08-21

**Authors:** Michiharu Sakamoto, Shuichi Ogino, Yoshihiro Shimizu, Masukazu Inoie, Sunghee Lee, Hiroki Yamanaka, Itaru Tsuge, Susumu Saito, Naoki Morimoto

**Affiliations:** 1 Department of Plastic and Reconstructive Surgery, Graduate School of Medicine, Kyoto University, Kyoto, Japan; 2 Department of Plastic and Reconstructive Surgery, Shiga University of Medical Science, Otsu, Japan; 3 Japan Tissue Engineering, Co., Ltd., Gamagori, Japan; Michigan Technological University, UNITED STATES

## Abstract

Allogeneic cultured epidermis (allo-CE) is a cultured keratinocyte sheet manufactured from donor cells and promotes wound healing when used in deep dermal burns, donor sites, and chronic ulcers and serves as a wound dressing. Allo-CE is usually cryopreserved to be ready to use. However, the cryopreservation procedure will damage the cell viability, and the influence of Allo-CE, according to its viability or wound healing process, has not been evaluated sufficiently. In this study, we aimed to prove the influence of keratinocyte viability contained in allo-CEs on wound healing. We prepared CEs with Green’s method using keratinocytes obtained from a polydactyly patient and then prepared four kinds of CEs with different cell viabilities [fresh, cryopreserved, frozen, and FT (freeze and thaw)]. The cell viabilities of fresh, cryopreserved, frozen, and FT CEs were 95.7%, 59.9%, 16.7%, and 0.0%, respectively. The four CEs had homogeneous characteristics, except for small gaps found in the FT sheet by transmission electron microscopy observation. The four CEs were applied on the full-thickness skin defect of diabetic mice (BKS.Cg-Dock 7^m^ +/+ Lepr^db^/Jcl), and the wound area and neoepithelium length were evaluated on days 4, 7, and 14. As a result, FT CEs without viable cells similarly promoted epithelialization on days 4 and 7 (p<0.05) and accelerated wound closure on day 7 (p<0.01) as fresh CEs compared with the control group. In conclusion, the promoting effect of allo-CE on wound healing does not depend on cell viability. Lyophilized CEs may be a suitable wound dressing with a long storage period at room temperature.

## Introduction

Cultured epidermal autograft (CEA) is a cultured keratinocyte sheet manufactured from the patient’s skin biopsy and is effective in the treatment of severe burns [1,2]. However, a delay of 3–4 weeks required for the generation of a CEA from the patient’s skin creates a serious clinical problem because it occurs during the life-threatening early phase in the treatment of severe burns. Besides, the clinical use of CEA has limitations because of lower take rate (“take” means that applied CEA survives on the wound and regenerate epidermis here), mechanical fragility, and frequent spontaneous blistering, particularly at the early stages [3].

Meanwhile, allogeneic cultured epidermis (allo-CE) is a cultured keratinocyte sheet manufactured from donor cells; therefore, it can be initially prepared and stored to be applicable immediately after a burn injury. Allo-CE promotes wound healing when used in deep dermal burns [4–8], donor sites [9–12], and chronic ulcers [13–15] and serves as a wound dressing. With allo-CE, it does not survive on the wound surface to generate epidermis because of the recipient’s immune response, and the wound would heal beneath the applied allo-CE by the epithelialization of recipient cells in the appendixes in the wound bed or wound edge [16].

In Korea, cryopreserved allo-CE product (Kaloderm^®^, Tego Science, Gangseo-gu, Korea) is already approved and available for burn wound treatment. Similar to this Korean product, allo-CE is usually cryopreserved and stored in hospitals and burn centers to keep them available for burn patients.

However, the cryopreservation procedure can damage the cells and tissues, reducing cell viability. The cell viability in cryopreserved CEs is depleted after the cryopreservation procedure [17]. An important unsolved issue is how much the efficacy for wound healing promotion is deteriorated by the depletion of cell viability in cryopreserved allo-CE. To elucidate this issue, we prepared several kinds of CEs with different cell viabilities. Then, we compared wound healing using mouse full-thickness skin defect model. In this study, we aimed to prove the influence of cell viability of keratinocytes constituting allo-CEs on wound healing.

## Materials and methods

### Ethics statement

In this study, the keratinocytes used to prepare the CEs were obtained from the skin of a supernumerary finger resected from a polydactyly patient at Kyoto University Hospital. The donor’s parents provided written informed consent before the specimens were obtained. This protocol was approved by the Ethics Committee of Kyoto University Graduate School and Faculty of Medicine (Permit number R0467). Regarding animal research, our experimental protocol was approved by the Animal Research Committee of Kyoto University Graduate School of Medicine (Permit number Med Kyo 15150). The number of animals used in this study was kept to a minimum, and all possible efforts were made to reduce their suffering in compliance with the protocols established by the Animal Research Committee.

### CE preparation

Keratinocytes were obtained from the skin of supernumerary finger resected from a 1-year-old boy with polydactyly using Green’s method as described previously [18,19] with some modifications. Briefly, the subcutaneous tissue of the harvested skin was removed with scissors, and the remaining skin was minced to pieces less than 1 mm in diameter. After trypsinization, the keratinocytes were isolated from the supernatant, disseminated on irradiated 3T3-J2 cells used as a feeder layer in a flask (TPP tissue culture flask, Sigma-Aldrich Japan, Tokyo), and cultured in Dulbecco Modified Eagle Medium (DMEM) and Ham’s F12 medium mixed 3:1. It supplemented with 5% fetal calf serum, insulin, hydrocortisone, cholera toxin, triiodothyronine, epidermal growth factor, and antibiotics in an atmosphere of 10% CO_2_ at 37 °C. Thereafter, the proliferated keratinocytes were collected and cryopreserved with glycerol until the time for use. To prepare CEs, the cryopreserved cells were thawed at 37 °C and cultivated about one week to confluence. The keratinocytes were cultured in the culture medium with conventional technique without exposure to the air phase.

The CE was obtained as keratinocyte sheets, which were detached from flasks after treatment with dispase (dispase II, FUJIFILM Wako Chemicals, Osaka, Japan) and then backed with non-woven gauze made of cellulose for carrier of 10 x 8 cm in size for easy handling. Precisely, after treatment with dispase according to the manufacturer’s protocol, a carrier sheet was put on the cultured keratinocytes in the flask, and the edge of the keratinocyte sheet was turned up on the carrier sheet.

By applying different freezing procedures to the prepared CEs, we prepared four kinds of CE with varying cell viabilities as follows:

1. Fresh: CEs kept at 20–28 °C without cryopreservation and used within 48 h after harvest from flasks.
2. Cryopreserved: CEs backed with carrier sheet were cut into 5 × 8 cm, put in a cryotube (Nunc CryoTube Vials #337516, Sigma-Aldrich Japan K.K., Tokyo, Japan) filled with 4.0 ml of cryopreservation medium containing 10% of dimethyl sulfoxide (DMSO) (STEM-CELLBANKER, Takara Bio Inc., Kusatsu, Japan), and cryopreserved at -80 °C for at least 7 days. The cryopreserved CE was thawed just before use by incubating at 37 °C for 7 min in a water bath, followed by gentle washing twice with normal saline.
3. Frozen: CEs backed with carrier sheet were cut into 5 × 8 cm, put in a cryotube filled with 4.0 ml of DMEM without DMSO, and stored at -80 °C for at least 7 days. When used, frozen CE was thawed and washed in the same method as cryopreserved CE.
4. Freeze and thaw (FT): CEs with carrier sheet kept in a plastic package filled with DMEM was kept at -80 °C for at least 120 min at first and then thawed by incubation at 37 °C in a water bath for 30 min. The freeze-thaw cycle was repeated five times to destroy cell membranes.

For histological assessment, the four CEs were fixed in 10% neutral-buffered formalin solution and embedded in paraffin to prepare 5-μm sections. The paraffin-embedded sections were then stained with hematoxylin-eosin (HE) and observed under an optical microscope (KEYENCE BZ-9000 and BZ-II Analyzer ver. 1.42).

For electron microscopic analysis, the four CEs were fixed in 0.1 M phosphate buffer with 2% glutaraldehyde and 4% paraformaldehyde at 4 °C overnight. After post-fixation with 1% OsO_4_ for 2 h, they were dehydrated and dried. For scanning electron microscopy (SEM), they were coated with a thin layer of platinum palladium. The specimens were examined with a Hitachi S-4700 scanning electron microscope (Hitachi, Tokyo, Japan). For transmission electron microscopy (TEM), thin sections were stained with 2% saturated uranyl acetate solution and 2.5% lead citrate solution and observed with a Hitachi H-7650 electron microscope (Hitachi, Tokyo, Japan).

### Cell viability of keratinocytes in CE

The cell viability of the four CEs was investigated. Each CE was placed into a 50-ml centrifuge tube containing 40 ml of ethylenediaminetetraacetic acid with 0.1% trypsin. The tube was incubated at 37 °C in a water bath until isolated. Then, DMEM with fetal bovine serum (FBS) was added to the tube to stop the enzyme reaction. The tubes were centrifuged at 1,000 rpm for 5 min and then resuspended with DMEM with FBS. Part of the cell suspension was mixed with an equivalent amount of 0.4w/v% trypan blue solution (Wako Pure Chemical Industries Ltd., Osaka, Japan), and the number of viable and non-viable cells were counted under a phase-contrast microscope (ECLIPSE TS100, Nikon, Tokyo, Japan).

### Application of CEs on the skin defects of diabetic mice

A total of 65 diabetic mice (BKS.Cg-Dock 7^m^ +/+ Lepr^db^/Jcl, 10-week-old male, CLEA Japan, Inc., Tokyo, Japan) were acclimatized in individual cages for 1 week before treatment. They had *ad libitum* access to sterile regular chow and water. Their body weight and blood sugar level were checked at two time points (1 week before the treatment and just before the treatment). Only mice with a high blood sugar level of over 350 mg/dl were used in this study. The eligible mice were allocated into five groups (control, fresh, cryopreserved, frozen, and FT groups; 13 mice in each group) so that the variance of the bodyweight of the five groups would be minimized. All painful treatments were performed under general anesthesia with inhalation of isoflurane. Isoflurane was given at 5% for induction and at 1–1.5% for maintenance. The entire dorsum of the animals was clipped and depilated with a depilation cream. Two full-thickness skin defects measuring 8 mm in diameter and in symmetrical position were created on both sides of the dorsum of each mouse. The wounds were 16 mm apart to avoid mutual interference. Each wound was covered with a respective CE (1.5 × 1.5 cm) in the Fresh, Cryopreserved, Frozen, and FT groups. Then, in all groups including the control group, the wounds were covered with non-adherent flexible contact layer (UrgoTul^®^, Urgo Laboratories, Leicestershire, UK) and polyethylene films containing absorbent cotton (Derma-Aid^®^; ALCARE Co., Ltd., Tokyo, Japan) to keep the wounds in a moist environment, after which the site was fixed with a surgical bandage (Silkytex^®^, Alcare Co., Ltd., Tokyo, Japan). The mice were checked daily to keep the bandage secured. For electron microscopic analysis, one mouse in each group was sacrificed on day 2 after the operation by inhalation of CO_2_, and the wounds were harvested. For TEM, thin sections were stained with the same method described above and observed with a Hitachi H-7650 electron microscope.

On days 4, 7, and 14, four mice in each group (eight wounds) were sacrificed by inhalation of CO_2_; digital photographs were taken to evaluate the wound area size. Then, the wounds were harvested and fixed in 10% neutral-buffered formalin solution. The specimens were embedded in paraffin to prepare 5-μm sections, which were then subjected to HE and immunochemical staining. Day 4 sections were stained with STEM121 to distinguish the transplanted human keratinocytes from recipient murine epidermis. STEM121 reacts specifically with a cytoplasmic protein of human cells. This marker is expressed in cells from tissues, including brain, liver, and pancreas. This antibody does not cross-react with tissues or extracts from mice and rats.

For the immunochemical staining of STEM121, paraffin-embedded sections were deparaffinized and rehydrated. Anti-STEM121 mouse monoclonal antibodies (dilution 1:1,500, code Y40410 Cellartis, Takara Bio Inc., Kusatsu, Japan) were used as primary antibody. Then, Histofine^®^ Simple Stain^™^ Mouse MAX-PO (Nichirei Biosciences Inc., Tokyo, Japan) was applied as secondary antibody and exposed to DAB (3–3’-diaminobenzidine-4HCl) (Nichirei Biosciences Inc.), and counterstaining was performed with hematoxylin.

### Assessment of the wound area and epithelialization

The wound area was measured using ImageJ ver. 1.45 (NIH, Maryland, USA) and is shown as the percentage relative to the original wound area. The length of the neoepithelium was determined by measuring the length from the wound edge to the end of the epithelium on HE-stained sections using an optical microscope (KEYENCE BZ-9000 and BZ-II Analyzer ver. 1.42, KEYENCE Japan, Osaka, Japan).

### Statistical analysis

Statistical significance was identified based on the Tukey-Kramer test. All data are expressed as the mean ± standard deviation. A P value of <0.05 was accepted as being statistically significant.

## Results and discussion

### Cell viability of keratinocytes in the four kinds of CE

**Fig 1:**
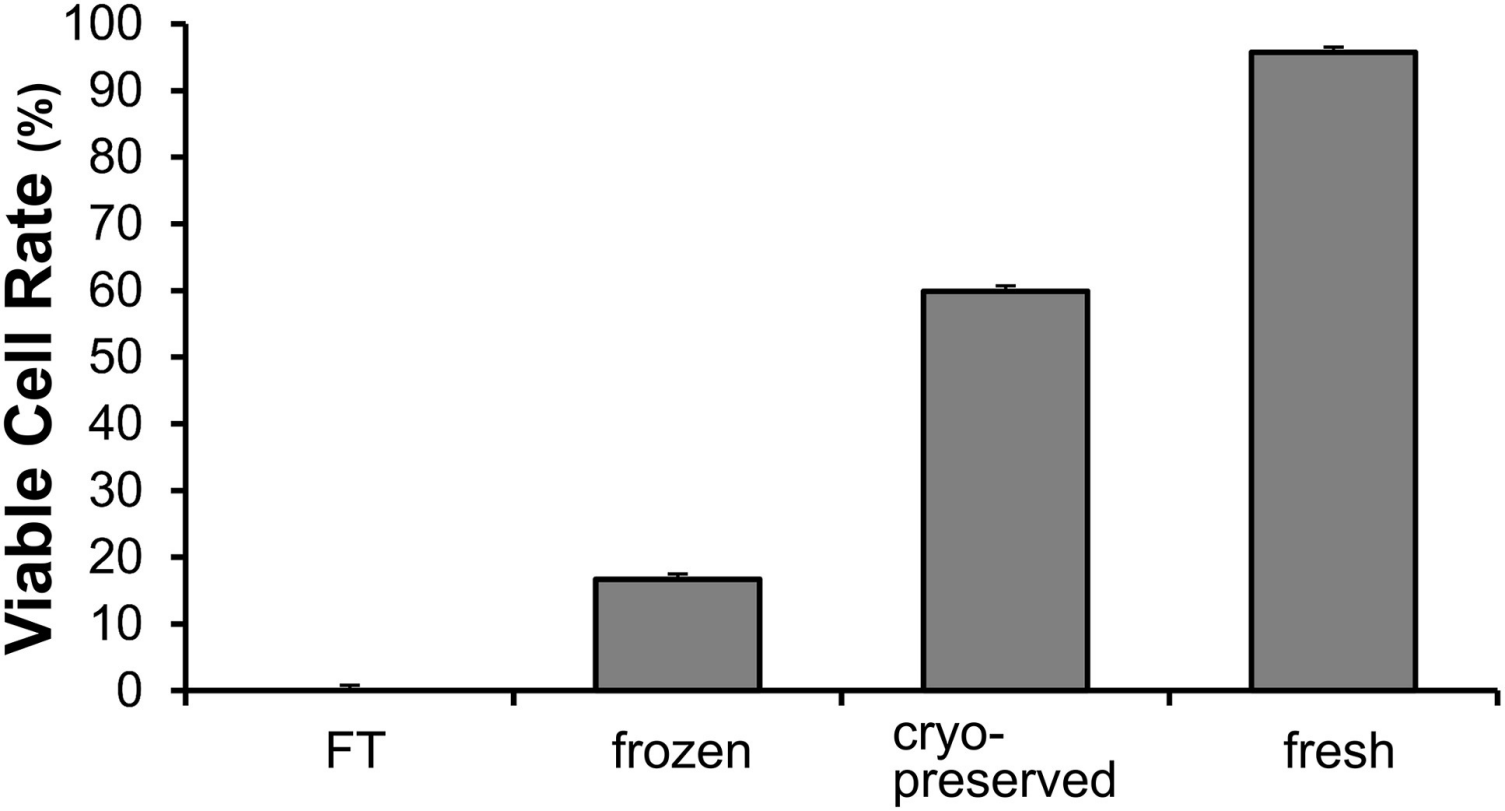
Cell viability of keratinocytes in the prepared cultured epidermis. Fresh, cultured epidermis without cryopreservation and used within 48 h; cryopreserved, cryopreserved at -80 °C for 7 days with DMSO; frozen, stored at -80 °C for 7 days without DMSO; and freeze and thaw (FT), the freeze-thaw cycle was repeated five times to destroy cell membranes.

The cell viabilities of fresh, cryopreserved, frozen, and FT CEs were 95.7%, 59.9%, 16.7%, and 0.0%, respectively (Fig 1). This procedure enabled us to prepare four kinds of CEs of different cell viabilities. Fresh CEs were composed of living cells, whereas FT CEs did not contain living cells. In the following experiment, these four CEs were used.

### Morphological assessment of four kinds of CE

**Fig 2:**
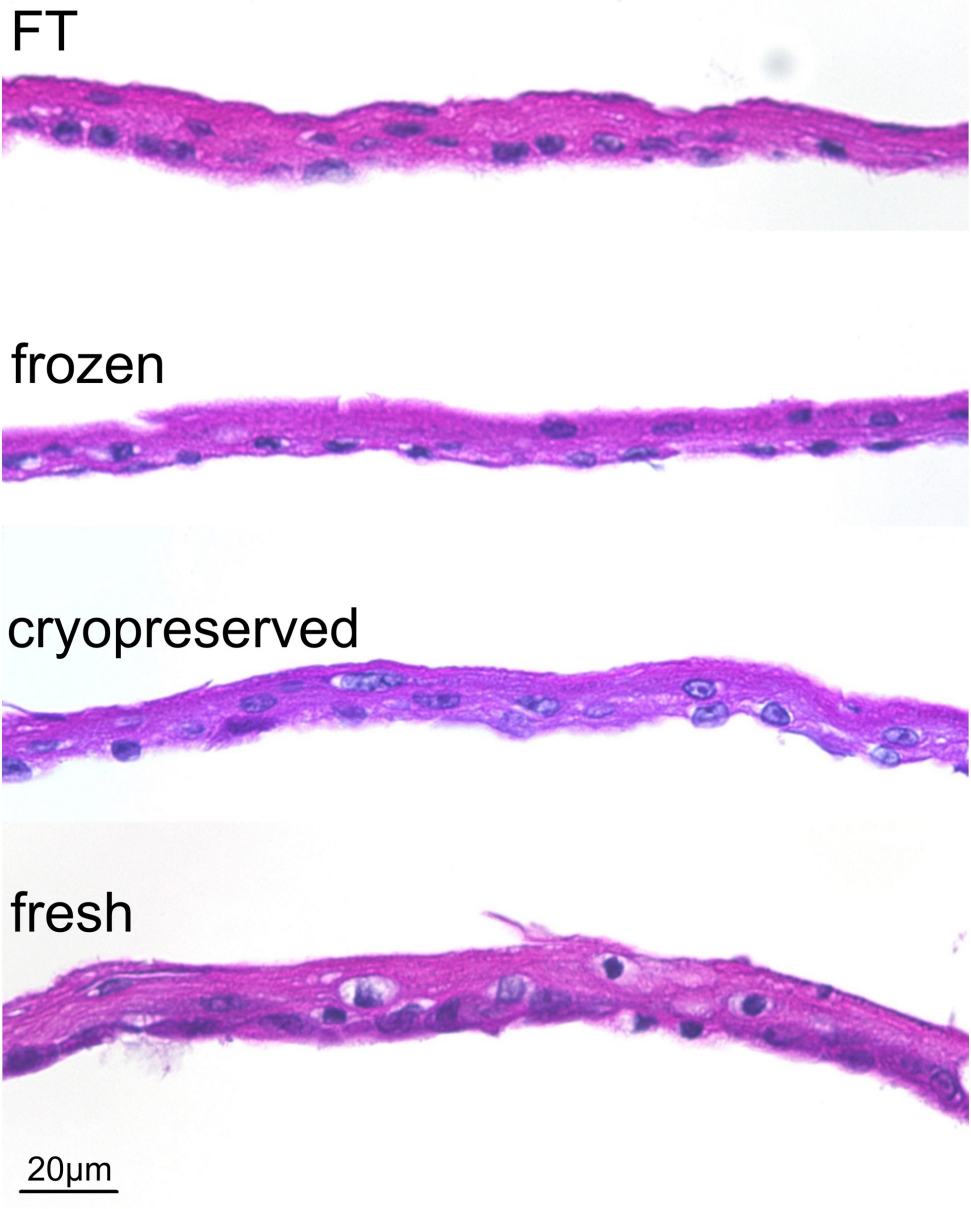
Cultured epidermis stained with hematoxylin and eosin. All CEs had similar morphological features, that is, consisting of 4–5 layers of keratinocytes, including the monolayer basal cell, constituting an undamaged membrane structure. FT, freeze, and thaw.

**Fig 3:**
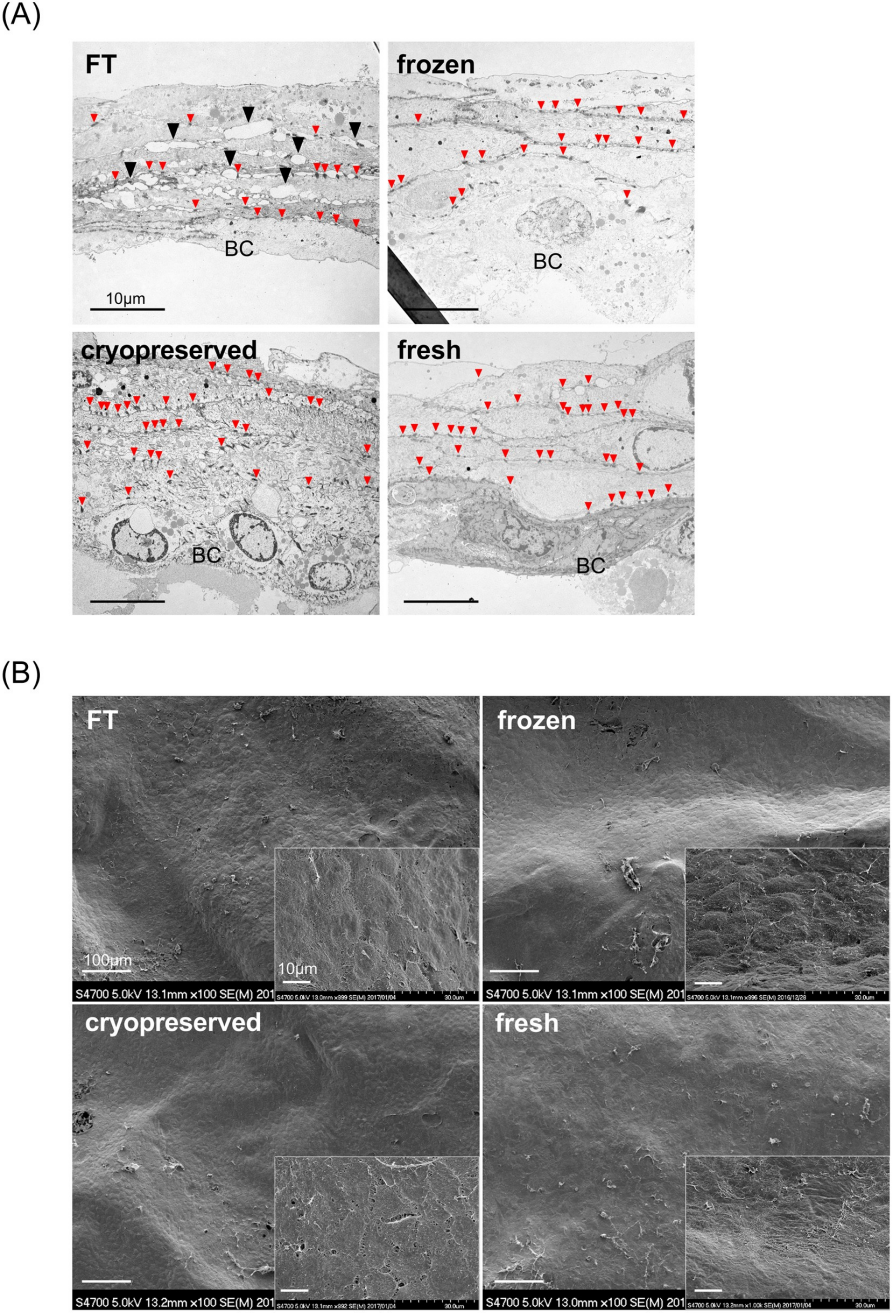
Electron microscopic survey of prepared cultured epidermis. A: Transmission electron microscopy (TEM): 5–6 layers of keratinocytes, including the monolayer basal cell and desmosomal complexes (small red arrowheads) at cell-contact points, were observed in all CEs. Intercellular adhesion was rigid in fresh, cryopreserved, and frozen CEs compared with that in FT. FT: numerous small gaps were found at the intercellular space, which indicates cell membrane injury (▲). BC: basal cell. B: Scanning electron microscopy (SEM): all CEs had a similar appearance, i.e., the keratinocytes formed a smooth membrane with regularity. No crack was found in the membrane surface, even in the FT sheet. FT, freeze, and thaw.

All CEs had macroscopically similar appearance, fragility, and handling characteristics. In HE-stained sections (Fig 2), all CEs had completely similar morphological features, that is, consisting of 4–5 layers of keratinocytes, including monolayer of basal cell, constituting an undamaged membrane structure. Nuclei shapes stained with hematoxylin were clearly confirmed with no metachromasy. In TEM studies (Fig 3A), as with HE section observation, 5–6 layers of keratinocytes, including the monolayer of basal cell and desmosomal complexes at cell-contact points, were clearly observed in all CEs. Intercellular adhesion was rigid in fresh, cryopreserved, and frozen CEs compared with that in FT. In FT CEs, numerous small gaps were found at the intercellular space, which indicates cell membrane injury. In SEM studies (Fig 3B), all CEs had a similar appearance, i.e., the keratinocytes formed a smooth membrane with regularity. No crack was found in the membrane surface even in the FT sheet.

### Wound healing in full-thickness skin defect model of diabetic mice

#### Wound size

**Fig 4:**
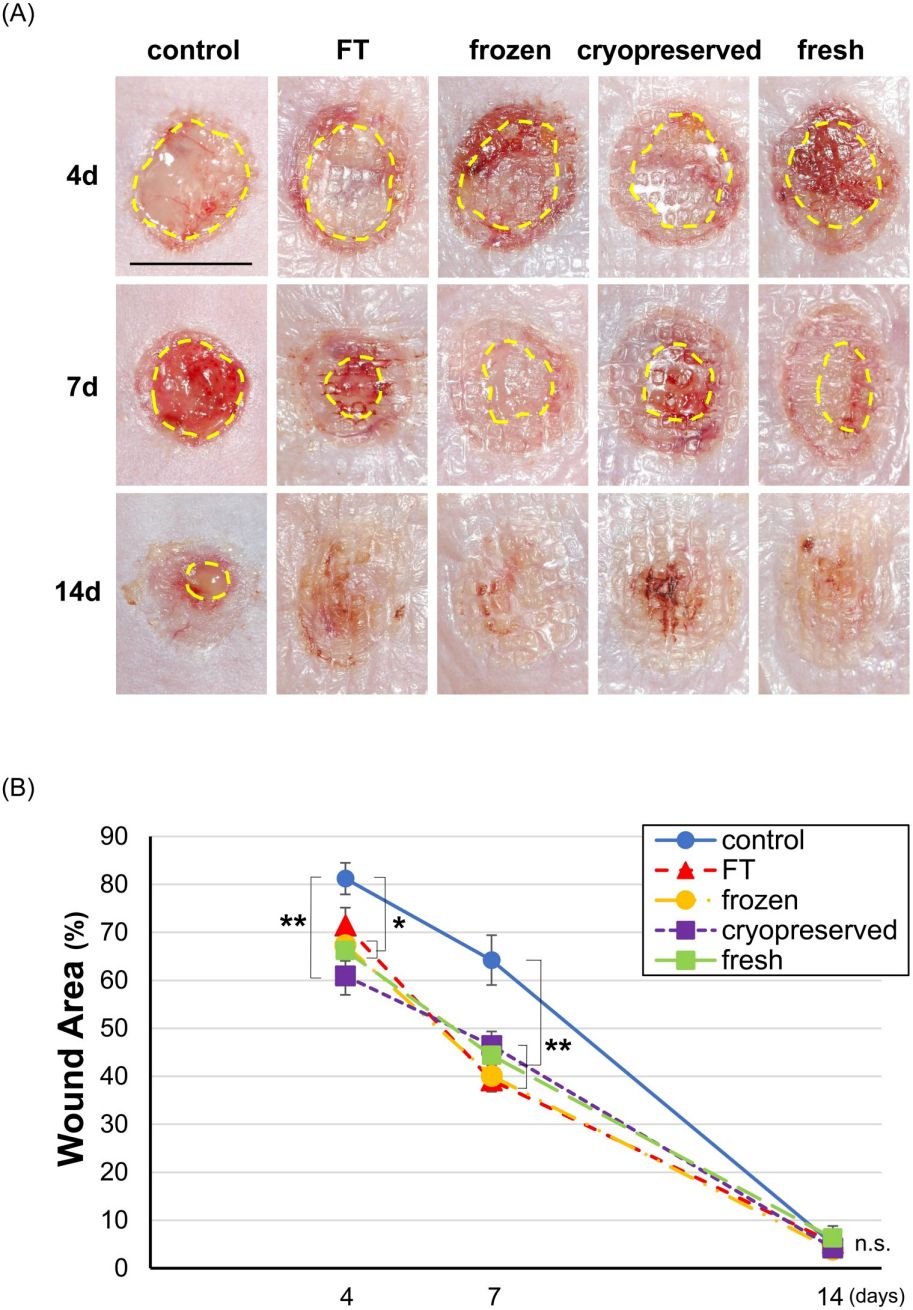
Wound area on days 4, 7, and 14 in the control, FT, frozen, cryopreserved, and fresh groups. A: The wound area was indicated by yellow-dotted lines. The applied CE is visible as a translucent membrane on the wound surfaces in the four CE groups (fresh, cryopreserved, frozen, and FT). No difference was observed in the appearance of the wounds among the four CE groups. Scale bar: 8 mm. B: Each wound area in the four CE groups was significantly smaller than that in the control group on day 7. (*p<0.05, **p<0.01, n = 8) FT, freeze and thaw.

The wounds on days 4, 7, and 14 are shown in Fig 4A. The applied CE is visible as a translucent membrane on the wound surfaces in the four CE groups (fresh, cryopreserved, frozen, and FT). No obvious infection was found in all groups at all time points. No difference was observed in the appearance of the wounds among the four CE groups. The wound area measured from digital photographs is shown in Fig 4B. At 7 days after the operation, each of the wound area in the four CE groups was significantly smaller than that in the control group (p<0.01, control vs. each of CE groups, n = 8), which means that all CEs similarly enhanced wound closure. At 14 days after the operation, the wound was almost closed in all groups, which indicated the fact that CE application did not cause any retardation on wound healing by immunologically induced inflammation or bacterial infection.

#### Neoepithelium length

**Fig 5:**
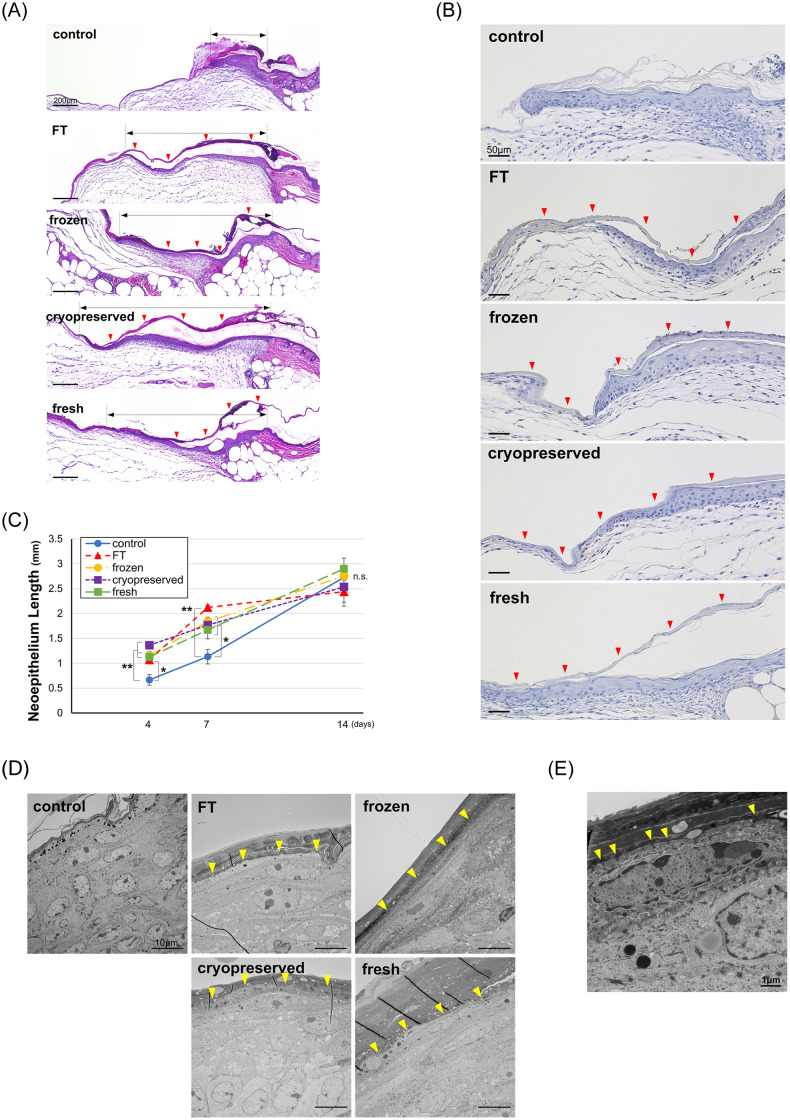
Wound healing effect of CEs on skin defect model of diabetic mice. A. HE-stained section on day 4 in the control, FT, frozen, cryopreserved, and fresh group. Red arrowheads indicate the applied CEs, and black arrows indicate the neoepithelium length. The newly formed epithelium in the control group was short and thick and composed of multiple layers of keratinocytes, whereas the four CE groups showed long and thin and composed of only a few layers of keratinocytes. B. Immunohistochemically stained sections with STEM121 in the control, FT, frozen, cryopreserved and fresh group on day 4. Red arrow heads indicate the applied CEs. The human CE attached to the wound surface completely, and recipient keratinocytes migrated from the wound edge beneath the CE and constituted an epidermis; then, the applied CE was detached from the keratinized surface of newly formed epidermis in the healed area. C. Neoepithelium lengths on days 4, 7, and 14 in the control, FT, frozen, cryopreserved, and fresh groups. Each of the neoepithelium length in the four CE groups was significantly larger than that in the control group on days 4 and 7. (*p<0.05, **p<0.01, n = 8). D. Transmission electron microscopy images on day 2 in the control, FT, frozen, cryopreserved, and fresh groups. Yellow arrowheads indicate the junction between the applied CE and murine keratinocytes. The transplanted human CE and cell membrane of mouse keratinocytes completely adhered without any gap. E. High magnification image of TEM on day 2 in the fresh group. The cell membranes of CE and mouse keratinocytes interlocked through its protrusions (yellow arrowheads). FT, freeze and thaw; CE, cultured epidermis.

The HE-stained sections on day 4 are shown in Fig 5A, and the immunohistochemically stained sections with anti-STEM121 antibody are shown in Fig 5B. In HE-stained sections, the newly formed epithelium in the control group was short and thick and composed of multiple layers of keratinocytes. On the contrary, the newly formed epithelia in the CE groups were long and thin and composed of only a few layers of keratinocytes. In the STEM121-stained sections, transplanted human CEs were stained with DAB to look brown; therefore, transplanted CEs could be distinguished from recipient keratinocytes. The transplanted human CE adhered to the wound surface without any gaps, and recipient keratinocytes migrated from the wound edge beneath the CE and constituted an epidermis; then, the applied CE was detached from the keratinized surface of the newly formed epidermis in the healed area.

The epithelium length is shown in Fig 5C. The epithelium lengths of the four CE groups were significantly larger than that of the control group at 4 and 7 days after the operation (day 4: p<0.01, control vs. fresh, cryopreserved and frozen, p<0.05, control vs FT; day 7: p<0.01, control vs FT, p<0.05, control vs fresh, cryopreserved and frozen; n = 8), which means that all kinds of CEs enhanced the epithelialization. Besides, no significant difference was observed among the four CE groups. TEM images of each group on day 2 are shown in Fig 5D. In all CE groups, the transplanted human CE and the cell membrane of mouse keratinocytes completely adhered without any gap. In the high magnification image (Fig 5E), cell membranes of CE and mouse keratinocyte interlocked through its protrusions, which indicated that the CE membrane adheres and covers the wound surface with cell-cell adhesion.

Allo-CE enhances wound healing and serves as a temporary biological dressing that releases many kinds of proteins, such as growth factors and basal membrane components [4,20]. Allo-CE needs to be prepared in advance and stored so that it is ready to use in the treatment of severely burned patients in the acute phase, while autologous CE is being prepared. For allo-CE to be readily available, different methods of preserving them for a substantial period of time without compromising their biological activities have been developed. Although the consistency of allo-CE was preserved by being stored in culture medium at 22–24 °C for 20 days [17] or cryopreserved at -80 °C in culture medium containing fetal calf serum and glycerol as a cryoprotectant [6], the cell viability of the keratinocytes was depleted in any case. When cells and tissues are cryopreserved, they are damaged by vitrification, cold shock, osmotic injury, and intracellular ice formation [21,22].

While there is a consensus that morphological aspects and biological characteristics should be retained in preserving allo-CE, the importance of cell viability has not been precisely investigated. Therefore, we studied the influence of cell viability of keratinocytes constituting allo-CE on its effectiveness to promote wound healing.

First, we prepared four kinds of CEs with different cell viabilities. Fresh CEs were unfrozen sheets and used as a positive control (95.7% viability). FT CEs were homogenized by freeze and thaw cycles until all cells were destroyed (0.0% viability). The freeze-thaw process damages the cell membrane by inducing ice crystal formation and/or oxidative stress [23]. DMSO is a common agent used as a cryoprotectant when cells or samples needed cryopreservation in a laboratory. When CEs were cryopreserved without any cryoprotectant, the cell viability declined to 16.7% (frozen CEs). DMSO prevented the depression of cell viability to 59.9% (cryopreserved CEs). All CEs were composed of 4–5 keratinocyte layers, and their microstructures determined by SEM were comparable. The membrane structure was preserved, and all CEs were applied to the mouse skin defects with no difficulty, although the damaged cell membrane in FT CEs was observed by TEM imaging. FT cycles resulted in small gaps at the intercellular spaces; however, they did not lead to through-holes or cracks penetrating all layers of CE.

Diabetic mouse (BKS.Cg-Dock 7^m^ +/+ Lepr^db^/Jcl) is a mutant strain that develops marked diabetic symptoms spontaneously, including obesity, overeating, and hyperinsulinemia, and widely used in diabetes research or studies on wound-healing [24,25]. This strain is immunocompetent and possesses the ability to develop an immune response. Therefore, the animal model applying human cell sheets used in this study was a xenograft model. The applied human keratinocytes on the mouse wound would be rejected by the recipient’s immune system and would not remain for a long time. Thus, the result of this study can be extrapolated to a clinical situation in which human CE is used as an allograft.

We applied four CEs with different cell viabilities on the full-thickness skin defects of mice, and evaluated wound healing on days 4, 7, and 14. As a result, all CEs accelerated epithelialization and wound closure on days 4 and 7, and no significant difference was found in the neoepithelium length and wound area among the four CE groups. This finding indicated that the accelerated re-epithelialization did not depend on the presence of proliferative human keratinocytes. Especially, in the FT group, freeze-thaw cycles completely damaged the cell membrane to stop cell activity, including protein production [23].

Many authors reported that the wound healing was promoted because allo-CE releases several growth factors and extracellular matrixes that stimulate the activity of the recipient’s cells at the application site [4,20]. However, if living cells are necessary to promote wound healing, the result of this study has no consistency. Biologically active substances stored in the keratinocytes, such as interleukin-1, vascular endothelial growth factor, and transforming growth factor-α, may be released from the keratinocytes regardless of cell viability and may contribute to wound healing [26]. Besides, some authors have mentioned that the membrane structure of the CE contributes to creating a comfortable environment for recipient cells in the wound bed to proliferate and migrate [17,20]. As HE-stained sections and TEM survey revealed, all CEs completely adhered to the wound surface without any gaps to cover the angulated surface of the exposed loose connective tissue at the wound bed. The applied CEs were also confirmed by immunostaining for STEM121 and distinguished from murine keratinocytes. The newly formed epidermis in CE-treated groups was very thin in the leading tip compared with that in the control group. Tamaritz et al. presented the same phenomenon and proposed that it occurs because the keratinocytes under CEs migrated faster than those in an open wound, resulting in fewer stratification [16]. We suppose that this phenomenon proves faster migration of keratinocytes.

In this study, we elucidated the fact that the mechanism of the promoting effect of allo-CEs on wound healing includes a factor that does not depend on the cell viability. When we suppose that dead CEs with no viable cells functions enough, there is no need to cryopreserve allo-CEs. For example, lyophilized CEs may be a suitable wound dressing, given its long storage period at room temperature. In fact, Jang et al. reported that lyophilized CEs accelerated wound healing at the same level as cryopreserved CEs [26]. Besides, CEs sterilized by gamma irradiation would have less probability of contamination (e.g., with bacteria and viruses) to be a much safer product.

We inferred that the promoting effect on wound healing of allo-CE does not depend on the cell viability; however, this finding is limited by the fact that this study was performed using the xenograft model. The keratinocytes in the CEs applied as allograft in the clinical setting may be possibly alive within a certain period [20,27]. In this case, the difference between living cell allo-CEs and deteriorated ones may cause a substantial difference in wound healing. Besides, the mechanism of wound healing promotion by deteriorated CEs was not fully proved in this study. Our next step would be to elucidate the mechanism of wound healing promotion by CEs without living cells and to perform a clinical study to make it available in clinical situations.

## Conclusions

Human CEs accelerated epithelialization and wound closure on days 4 and 7 after application in the full-thickness skin defect model of diabetic mice, but no significant difference was found among the CE groups with different cell viabilities. CEs with no viable cells, such as lyophilized CE would be a possible wound dressing material.
